# Mass Effect Deformation Heterogeneity (MEDH) on Gadolinium-contrast T1-weighted MRI is associated with decreased survival in patients with right cerebral hemisphere Glioblastoma: A feasibility study

**DOI:** 10.1038/s41598-018-37615-2

**Published:** 2019-02-04

**Authors:** Prateek Prasanna, Jhimli Mitra, Niha Beig, Ameya Nayate, Jay Patel, Soumya Ghose, Rajat Thawani, Sasan Partovi, Anant Madabhushi, Pallavi Tiwari

**Affiliations:** 1 Case Western Reserve University, Department of Biomedical Engineering, Cleveland, USA; 2 General Electric Global Research, New York, USA; 3 Department of Radiology, University Hospitals Cleveland Medical Center, Cleveland, USA

## Abstract

Subtle tissue deformations caused by mass-effect in Glioblastoma (GBM) are often not visually evident, and may cause neurological deficits, impacting survival. Radiomic features provide sub-visual quantitative measures to uncover disease characteristics. We present a new radiomic feature to capture mass effect-induced deformations in the brain on Gadolinium-contrast (Gd-C) T1w-MRI, and their impact on survival. Our rationale is that larger variations in deformation within functionally eloquent areas of the contralateral hemisphere are likely related to decreased survival. Displacements in the cortical and subcortical structures were measured by aligning the Gd-C T1w-MRI to a healthy atlas. The variance of deformation magnitudes was measured and defined as Mass Effect Deformation Heterogeneity (MEDH) within the brain structures. MEDH values were then correlated with overall-survival of 89 subjects on the discovery cohort, with tumors on the right (n = 41) and left (n = 48) cerebral hemispheres, and evaluated on a hold-out cohort (n = 49 subjects). On both cohorts, decreased survival time was found to be associated with increased MEDH in areas of language comprehension, social cognition, visual perception, emotion, somato-sensory, cognitive and motor-control functions, particularly in the memory areas in the left-hemisphere. Our results suggest that higher MEDH in functionally eloquent areas of the left-hemisphere due to GBM in the right-hemisphere may be associated with poor-survival.

## Introduction

Glioblastoma (GBM) is the most common primary brain tumor in adults and is characterized by a high proliferative rate and aggressive invasiveness in the brain^1^. Despite palliative therapy including surgical resection, radiotherapy, and chemotherapy, the treatment of GBM remains a challenge^2–4^. Unfortunately, the median survival in GBM patients is only 10 to 14 months after diagnosis^5,6^, with roughly 5–10% patients surviving for over 5-years. With new and promising experimental treatments (i.e. monoclonal antibodies, gene-, and immuno-therapies) currently under investigation, there is a need for new prognostic biomarkers for accurate risk stratification for personalized GBM management. Several studies previously have attempted to identify prognostic markers such as tumor size, location, treatment, age, Karnofsky performance score (KPS), and molecular markers (IDH, MGMT)^7–13^ for GBM survival stratification. Unfortunately, these markers have found limited clinical applicability, suggesting the need for identifying new non-invasive image-based prognostic markers towards improving GBM treatment management.

Magnetic Resonance Imaging (MRI) offers great utility as a standard-of-care protocol in diagnosis, grading, and management of GBM patients. Multi-parametric MRI (Gd-contrast T1w (Gd-T1w), T2w, FLAIR) offers the ability to visualize and quantify many of the physical manifestations of the pathologic processes in GBM^14–16^. For instance, enhancement on Gd-T1w MRI is known to be correlated with blood brain barrier (BBB) disruption, while T2w/FLAIR abnormalities are known to capture proliferative tumor margins and vasogenic edema^17^. Recently, quantitative imaging approaches termed as ‘radiomics’ have been used in conjunction with these routinely available imaging sequences to comprehensively characterize heterogenous tumors^18–21^. In the context of GBM characterization, these radiomics studies have involved extracting quantitative radiologic features (capturing co-occurrence, gray-level dependence, directional gradients, and shape-based measurements)^22–28^ and drawing associations of these features with clinical outcomes. The existing radiomics analysis however, has so far only been confined to computing intensity, and texture-based measurements within the tumor or in the peri-tumoral areas, to understand the effects of tumor proliferation on imaging with patient survival^23,26,28^. None of these studies have specifically attempted to analyze the changes on remote functional areas due to the growing tumor and surrounding mass effect (due to intracranial pressure).

For instance, midline shift is often caused in patients with mass effect i.e. the growing tumor mass and the edema pushing and displacing the surrounding brain structures^14^. Mass effect often results in alterations of consciousness, attention, and even awareness in a GBM patient. Further, mass effect in one hemisphere may demonstrate cognitive impairments suggesting damage to the contralateral hemisphere^29,30^. Chronic GBM symptoms such as headaches and seizures may often be non-focal and non-lateralizing to the tumor site due to increase in intracranial pressure^31^. Pre-surgery tissue displacement due to mass effect may also cause neurological deficits and hence play an integral role in predicting overall patient survival^12,32,33^. All of these chronic, debilitating symptoms, which negatively affect patient’s ability to function normally, may finally lead to a fatal outcome^34^. It may therefore be important to study the changes in parts of the brain, remote to the tumor location, that are impacted by the growing tumor within the confined environment of the brain vault.

Based on these observations, the scientific rationale of this study is that the mass effect due to growing tumor will have an impact on the contralateral cerebral hemispheric structures as observed on T1-weighted (T1w) imaging, and this impact in the cortical and sub-cortical structures will have negative implications on patient’s overall survival. We measure the tissue deformation due to the impact of mass effect in the cortical and sub-cortical structures, from the magnitudes of voxel-wise deformation fields obtained by aligning the GBM subject to a normal subject. We then compute variance observed in the tissue deformation and define this measure as Mass Effect Deformation Heterogeneity (MEDH). Our rationale is that MEDH across contralateral functional areas before surgery, as observed on T1w MRI, is an associated factor in overall survival of GBM patients.

The rest of the paper is organized as follows. Data cohort and methodology involving MEDH are detailed in the materials and methods section. A detailed description of our findings is reported in the results section. Subsequently we analyze our findings in the discussion section, followed by concluding remarks.

## Materials and Methods

### Participants

A total of 262 GBM MRI studies were obtained from the Cancer Imaging Archive (TCIA) database. TCIA is an open archive of cancer-specific medical images and associated clinical metadata established by the National Cancer Institute (NCI) and collaborating institutions in the United States. The Health Insurance Portability and Accountability Act (HIPPA) compliant project in the cancer genome atlas (TCGA) was conducted in compliance with regulations and policies for the protection of human subjects, and approvals by institutional review boards were appropriately obtained. The preoperative MRIs of the GBM subjects were made available for public download from TCIA. Previous studies have leveraged this dataset extensively for imaging-genomic mapping^35^ as well as radiomic analysis^36^.

The inclusion criteria for this study consisted of the following: (1) availability of all 3 routine pre-surgical MRI sequences (Gd-T1w, T2w, FLAIR) for treatment-naïve patients; (2) MRI scans with diagnostic image quality (as determined by consensus across 2 expert radiologists); (3) availability of overall patient survival information, age and KPS; (4) no errors post registration and (5) subjects with a single tumor lesion. Of the 262 studies, a total of 124 studies were excluded from analysis based on our inclusion criteria. Of the remaining 138 studies, a total of 89 GBM subjects (55 males, and 34 females) were used as discovery cohort^37^, while the remaining 49 subjects (31 males and 18 females) were used as hold-out validation cohort. TCIA IDs as well as the resection status of the 138 cases used in this work have been provided in the Supplementary Material S1.

In our discovery cohort, 41 subjects had right hemispheric tumors, and 48 subjects had left hemispheric tumors. In the validation cohort, 19 subjects had right hemispheric tumors, and 30 subjects had left hemispheric tumors. The KPS at presentation was between 36 and 100 with a mean of 77.96 ± 13.78 for the discovery set and 79.87 ± 13.27 for the validation cases, respectively. The overall survival of the patients was stratified into short-term (≤7 months), long-term (>18 months)^6,7^ and the remaining into medium-term (>7months to 18 months) survivors. The cutoff values used in our experiments were agreed upon by the expert neuro-radiologists in our group and were partially driven to provide the understanding of how deformations in specific functional areas impact extreme ends of GBM survival. Using the defined cutoff, we obtained a total of 38 short-term, 56 medium-term and 44 long-term survival cases, respectively. A summary of the patient clinical variables is given in Table 1 based on their survival groups. A total of 133 of the 138 cases had resection status available, out of which 125 cases had a gross total resection (93.9%), and the remaining had excisional biopsy or fine needle aspiration biopsy (6.02%). The patients underwent external beam radiation therapy on the primary tumor field, with a radiation dose of 4853 +/− 2053 cGy.

**Table 1:** Patient clinical variables based on overall survival (OS) in months as short (OS ≤ 7 months), medium (OS > 7months to 18 months), and long-term (OS > 18 months) survivors for the discovery and validation cohort.

	Discovery cohort			Hold-out validation cohort		
	Short	Medium	Long	Short	Medium	Long
Total	26	35	28	12	21	16
Right Tumor	12	17	12	3	6	10
Left Tumor	14	18	16	9	15	6
Right Tumor Vol (ml)	21.25 ± 17.45	17.50 ± 11.98	11.14 ± 9.60	44.39 ± 29.83	19.51 ± 15.51	39.22 ± 53.96
Left Tumor Vol (ml)	14.69 ± 15.41	10.83 ± 10.07	14.95 ± 11.31	80.63 ± 79.40	26.15 ± 19.23	24.48 ± 6.60
Male (%)	53.8	65.7	64.2	50	71.4	62.5
Median Age (yr)	66.5	59	55.5	60	57.5	58.5
Median KPS	80	80	80	80	80	80
Median Survival (mo)	4.03	12.06	25.56	3.03	11.1	29.14
Mean Survival (mo)	4.18	11.86	31.27	3.59	12.22	32.38

### Image acquisition

The TCIA MRI studies were acquired at different sites using either 1.5 Tesla or 3 Tesla GE (Signa Excite) or Philips (Gyroscan Intera) or Siemens (Trio, Avanto or Symphony) scanners. The post gadolinium contrast (Gd-C) T1w images used in this study were acquired using spin echo (SE) sequence for a majority of cases with only 2 cases using spoiled gradient recalled echo (SPGR). The T1w images from multiple scanners had TR/TE/TI as 500–1800/5.7-14/785-1238 ms, acquisition matrices of either 256 × 192 or 320 × 224 and voxel spacing ranging from 0.468 × 0.468 × 2.5 to 0.937 × 0.937 × 5, flip angles 15–90 degrees. The T2w images had TR/TE as 750-3766/25-104 ms, acquisition matrices of either 256 × 192 or 320 × 224 and voxel spacing ranging from 0.468 × 0.468 × 2.5 to 0.937 × 0.937 × 5, flip angles 20–90 degrees. The FLAIR images had TR/TE/TI as 700-10004/15-155/2100-2250 ms, acquisition matrices of either 256 × 192 or 320 × 224 and voxel spacing ranging from 0.468 × 0.468 × 2.5 to 0.937 × 0.937 × 5, flip angles 90–150 degrees.

### Expert segmentations and MRI reads

GBM lesions were evaluated on axial Gd-C T1-weighted (T1w), T2-weighted (T2w), and FLAIR MRI sequences. The lesions were manually delineated by an expert (S.P, 7 years of experience in radiology), who segmented every tumor lesion into necrotic core, active region, and edema. Tumor necrosis on Gd-T1w was identified as areas of relatively hypo-intense regions (occasionally with ring-enhancement) which is frequently located in the tumoral region. T2w and FLAIR scans were used to identify edematous regions, while necrosis and enhancing tumor were delineated based on post gadolinium T1w MRI. These annotations were further confirmed by a second expert radiologist (A.N, board certified with over 10 years of experience in neuro-radiology). The T1w and FLAIR scans, on the discovery cohort (n = 89), were further reviewed by the more experienced radiologist (A.N) for any observable radiological changes especially in the contra-tumoral hemisphere as midline shifts, tumors crossing the corpus callosum (CC), changes in contra-tumoral lateral ventricle (LV) and subfalcine or transtentorial herniation. Table 2 lists the observed radiological manifestations that impact the contralateral hemisphere.

**Table 2:** Radiological changes in contralateral tumor structures in n = 41 right-hemispheric and n = 48 left-hemispheric tumors as observed by an experienced neuroradiologist in the discovery cohort.

Tumor	Midline shift	Crossing CC	Deformed LV	Herniation	None
Right	33	12	11	5	6
Left	33	10	3	1	11

Midline shift was qualitatively evaluated, crossing over of tumor through corpus callosum (CC) was either through genu or splenium, contra-tumoral lateral ventricles (LV) were either narrowed or enlarged, herniation was primarily subfalcine. The first column is the cerebral hemisphere of the tumor and the last column shows the number of cases where no abnormality was observed by the radiologist.

### Data preprocessing

The T1w MRI images were corrected for intensity inhomogeneity induced by bias of the magnetic head coil using N4 bias-correction method^38^. As TCIA database consists of multicenter data with varied T1w intensities for each subject, the images were normalized using a histogram matching method (bins = 255, points = 64)^39^ to match the intensities of a normal T1w MNI (Montreal Neurological Institute) atlas. The subject brains were skull-stripped by non-rigidly aligning^40^ the T1w MNI atlas to each of the subject T1w images. The same transformations were then applied to the MNI brain mask to obtain patient-specific brain masks. The mapping was calculated as a piece-wise linear transform between corresponding ranges on the intensity distributions of the two acquisitions. Using the expert annotations, intensity values corresponding to enhancing tumor, necrotic core, and edema were excluded from influencing the intensity standardization procedure.

### Methodology

**Figure 1:**
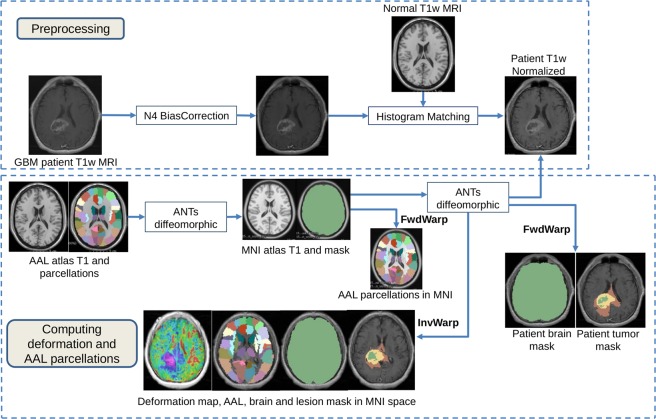
Schema diagram of our proposed method to compute deformation in brain tissues due to tumor mass effect. MNI = Montreal Neurological Institute, AAL = Automated Anatomical Labeling, ANTs = Advanced Normalization Tools.

Our approach consisted of two stages: (a) Computing voxel-wise tissue deformation and segmenting brain structures; and (b) Statistical analysis of deformation heterogeneity and overall GBM survival. A schematic diagram depicting the preprocessing and stage (a) is provided in Fig. 1.

#### Computing deformation due to mass effect using normal atlas

The T1w images of the GBM subjects were aligned to a healthy T1w MNI atlas in order to study the deformation of remote tissue structures due to tumor mass effect. A deformable registration involving the Advanced Normalization Tools (ANTs) Symmetric Normalization (SyN)^40^ method was employed. The SyN diffeomorphic registration available in ANTs toolkit has been known to be robust in mapping brain structures to a healthy template in presence of brain lesions^41–43^. ANTs efficiently handles the constrained cost-function masking approach where the mapping within a tumor exclusive region is determined by the solution of the negative tumor mask region. The combined tumor area including the necrotic core, active region, and edema was excluded in the computation of the similarity measures for deformable registration. This ensured exclusion of intensity differences within the tumor area; while only considering the spatial intensity differences due to structural deformation caused by mass effect when compared to the corresponding healthy atlas.

Healthy T1w MNI atlas was used to measure the tissue deformation in the normal appearing brain regions of every patient volume. The atlas was first non-rigidly aligned to the patient volume using mutual information based similarity measure provided in ANTs (Advanced Normalization Tools) SyN (Symmetric Normalization) toolbox. The tumor mask was removed from the patient volume during registration such that only the spatial intensity differences due to structural deformation caused by mass effect are recovered, when compared to the atlas. Given the reference patient volume and floating atlas, the non-rigid alignment can be formulated as T(Atlas) where, T(.) is the forward transformation of the composite (including affine components) voxel-wise deformation field that maps the displacements of the voxels between the reference and floating volumes. This transformation also propagates the atlas brain mask to the subject space, thereby skull-stripping the subjects. As ANTs SyN satisfies the conditions of a diffeomorphic registration, an inverse T’ exists, that successfully maps the patient volume to the atlas space. This inverse mapping yields the tissue deformation of the subject volume with respect to the atlas, representing the deformations exerted on every voxel, due to the tumor mass effect.

The voxel-wise deformation in each GBM subject was obtained as a deformation field-vector $(\overrightarrow{x},\overrightarrow{y},\overrightarrow{z})$ in three planar orientations. A Euclidean norm of the scalar values of the deformation orientations was computed as $\sqrt{{x}^{2}+{y}^{2}+{z}^{2}}$ to obtain the magnitude of deformation per voxel.

#### Extraction of mass effect deformation heterogeneity (MEDH) within functional areas

**Figure 2:**
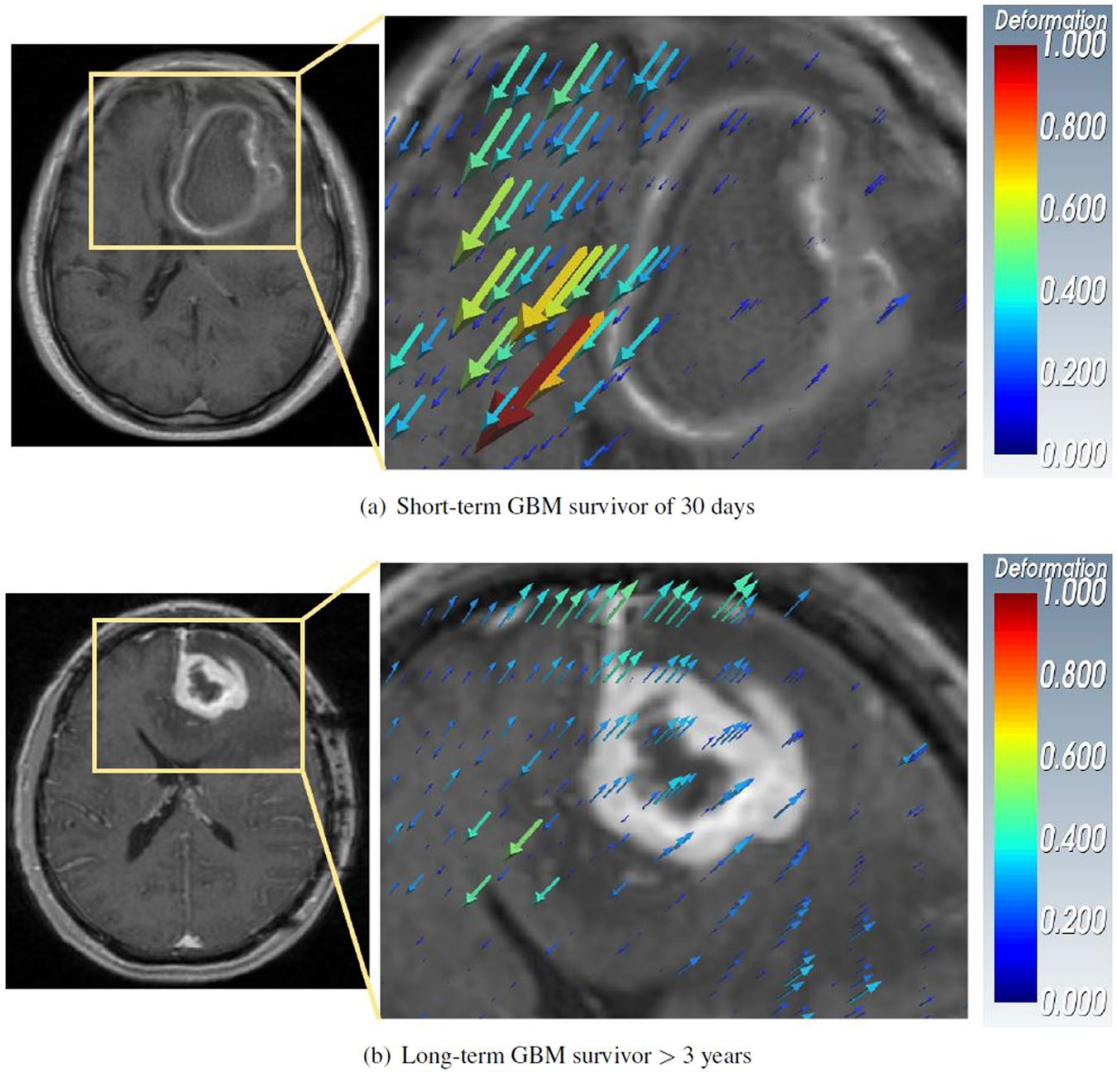
Deformation vectors representing tissue displacement are shown as volume rendered 3D quivers overlaid on an image slice of right-hemispheric GBMs. The deformation magnitude is proportional to the size of quivers. Higher value of deformation magnitude is represented by ‘red’ and lower value by ‘blue’ color respectively. The quivers also show the direction of tissue displacement. Neurological view is shown.

Figure 2 shows two cases of frontal right-hemispheric GBMs where a high variance in tissue deformation magnitude is observed around the tumor pushing into the contralateral hemisphere for a short-term survivor (30 days) in Fig. 2(a) when compared to a long-term survivor (>3 years) in Fig. 2(b). The precise difference in the varied degrees of deformation between the extreme survival cases in Fig. 2 allows the conception of the term Mass Effect Deformation Heterogeneity (MEDH). Specifically, MEDH is defined as the variance of per-voxel tissue displacement magnitudes due to mass effect in contralateral/ipsilateral functional areas that may be associated with GBM survival.

The Automated Anatomical Labeling (AAL)^44^ atlas with 116 regions parcellated as the anatomical structures including deep brain structures was used in our study, since we wanted to capture MEDH within functionally eloquent parts of the brain. The T1w atlas was registered non-rigidly to the T1w MNI atlas^40^ and the same transformation was applied to the AAL parcellations. Variance of voxel-wise deformation magnitudes within each of these anatomical structures was used as a measure of MEDH, and used for further survival analysis.

#### Associating MEDH with long and short-term survival on the discovery cohort

The statistical association between MEDH of anatomical structures and patient survival was performed separately for right-hemispheric and left-hemispheric tumors to tease out the effect of deformation due to tumor presence in contralateral hemisphere. As the tumor areas were excluded from the computation of similarity measures for deformable alignment (as explained in Section 2), the magnitudes of deformation within these areas were close to null. This means, for the group with right cerebral GBMs, the relevant deformations would be within the contralateral functional areas and in areas of the right hemisphere without any tumor overlap, and similarly deformations within the right-hemispheric functional areas would be pertinent for left cerebral GBMs.

The first set of statistical correlations, within the discovery cohort, were performed with the group of long-term and short-term survivors only, correlating MEDH within each of the 116 AAL regions with the total number of days of survival (as recorded from the baseline-scans). Spearman’s rank correlation^45^ was used, given that it is robust against non-parametric distribution of data^46^. The correlative analysis was performed on *n* = 12 short-term (mean: 106 ± 53 days) and *n* = 12 long-term (mean: 828 ± 272 days) survivors within the right cerebral tumor group. Similarly, the left-hemispheric tumor group with *n* = 14 short-term (mean: 142 ± 44 days) and *n* = 16 long-term (mean: 1018 ± 430 days) survivors was analyzed separately.

The second set of statistical correlations were performed between MEDH and GBM survival on the same sets of long- and short-term survivors and for the left- and right-hemispheric tumor groups separately, considering the total tumor volume (includes necrotic core, active region and edema) and age of the subjects as confounders. This was done to make sure that the known factors of the GBM survival were not impacting the analysis particularly as the median age was not matched between the long- and short-term survivors, and the mean right-hemispheric tumor volume was higher for the short-term survivors compared to the long-term survival group (see Table 1).

#### Analysis of MEDH versus survival in medium-term group on the discovery cohort

This analysis was performed specifically to understand which brain areas impacted due to mass effect were associated with GBM survival in general, without considering the extreme ends and only focusing on the medium-term survival group. Spearman’s rank correlations were performed for the medium-term survival groups for right-hemispheric (*n* = 17, mean: 364 ± 79 days) and left-hemispheric (*n* = 18, mean: 368 ± 92 days) tumors separately. A separate set of correlations was also performed considering the age and total tumor volume as confounding factors.

#### Validation of MEDH-survival correlation on the hold-out cohort

Validation comprised four different experiments within the hold-out cohort of n = 49 cases, involving associating MEDH values in (a) left-hemispheric AAL regions of right cerebral tumor cases with survival in long and short term survivors, (b) right-hemispheric AAL regions of right cerebral tumor cases with survival in long and short term survivors, (c) left-hemispheric AAL regions of right cerebral tumor cases with survival in medium term survivors, (d) right-hemispheric AAL regions of right cerebral tumor cases with survival in medium-term survivors, similar to the experiments performed for the training cohort. Further, a Cox proportional hazards survival model was built to obtain concordance indices (CI or C statistic) for each of the different AAL regions that were most correlated with the survival days. CI is the fraction of all pairs of subjects whose predicted survival times are correctly ordered (i.e. concordant with actual survival times). CI = 1 indicates that the model has perfect predictive accuracy, and CI = 0.5 indicates that the model is not better than random chance. A multi-variate analysis by combining all the significant AAL regions within the Cox proportional hazard model was also performed.

### Software implementation

All preprocessing steps were performed using ITK (www.itk.org) based implementations. The image processing and deformation analysis for the patients were performed in a parallel computing environment utilizing the Case Western Reserve University High Performance Computing Cluster. FSLUtils tool of FSL^47^ (fsl.fmrib.ox.ac.uk/fsl/fslwiki/Fslutils) was used to compute the variance of voxel-wise deformation magnitudes. The Spearman correlations were performed using the Python Scipy package (www.scipy.org). The second set of correlations that involved computation of partial correlations for age and tumor volume as confounding factors, and computation of C indices were performed using the R statistical package (www.r-project.org). All figures were created using 3D Slicer (www.slicer.org) and Smili (github.com/shakes76/smili).

## Results

### Association of MEDH with GBM survival

**Figure 3:**
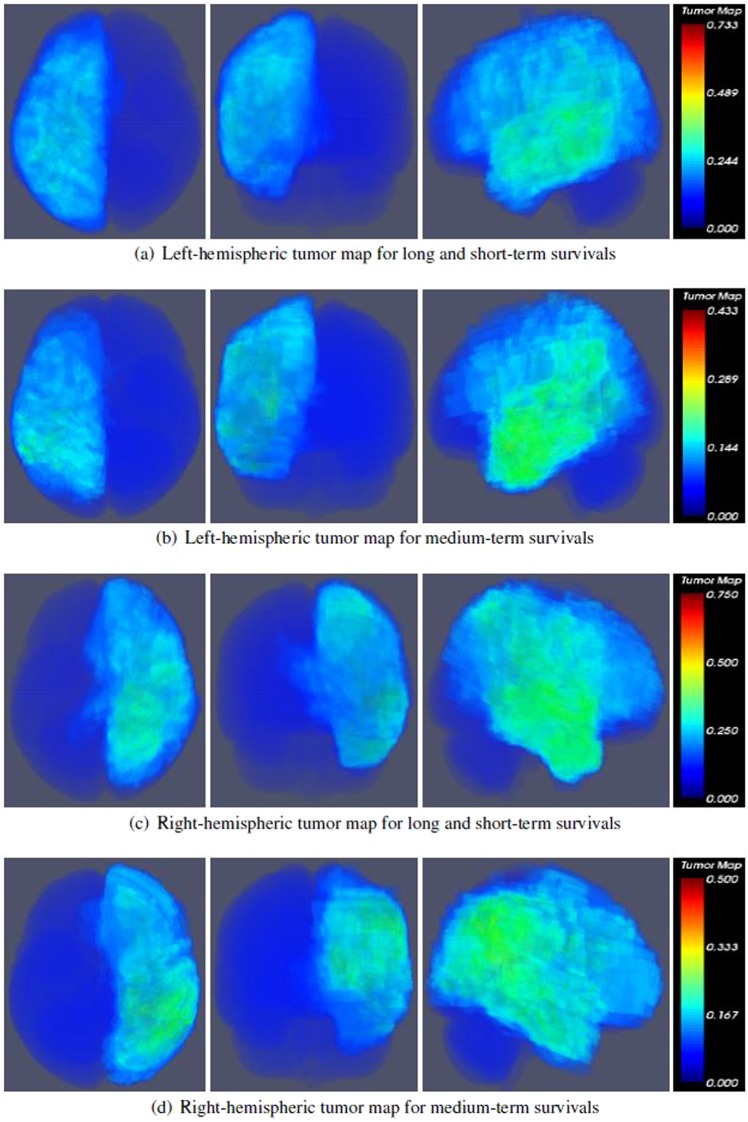
Average tumor overlap for right and left-hemispheric tumors for different groups of patients based on survival (neurological view). The tumor maps are shown in axial, coronal and sagittal views and the color maps show the probability of tumor between 0 and 1.

We observed that MEDH in several functional areas was negatively correlated (*p* < 0.05) with survival across both hemispheres for the right hemispheric tumor group. Although, significant correlations were found for AAL regions in both hemispheres, we believe the structures in the contralateral hemisphere were more relevant for the analysis as the structures of ipsilateral hemisphere may overlap with the tumor representation for the group. The tumor maps for the right and left-hemispheric groups shown in Fig. 3 suggest a large degree of midline shift for the right-hemispheric tumors compared to the left-hemispheric tumors. Hence, for right-hemispheric tumors, the MEDH in contralateral functional areas appear to be significantly associated with long, short and medium-term survival. Interestingly, no significant association was found across any AAL region and survival group for MEDH of left-hemispheric tumors. The significant correlation values for the AAL regions of the right cerebral tumor group for both discovery (*r*_*dis*_) and validation sets (*r*_*val*_), across long- and short-term survival groups are presented in Table 3. *r*_*dis*_ values, with age and tumor volume as confounders, are presented in Table 4 for the long- and short-term survival. The significant correlation values for the AAL regions of the right cerebral tumor group for both discovery and validation sets are presented in Table 5 for the medium-term survival group. Similarly, *r*_*dis*_ values, with age and tumor volume as confounders, are presented in Table 6 for the medium-term survival group.

**Table 3:** AAL regions of the right cerebral tumor groups, within which MEDH was negatively correlated with long and short-term survival.

Left Hemisphere				Right Hemisphere			
Region	*r*_*dis*_	*p*	*r*_*val*_	Region	*r*_*dis*_	*p*	*r*_*val*_
Hippocampus	−0.43	0.034	−0.21	Olfactory Cortex	−0.41	0.045	−0.29
Lingual_G	−0.47	0.018	−0.23	Insula	−0.43	0.034	−0.11
Postcentral_G	−0.42	0.036	−0.44	Parahippocampal_G	−0.50	0.011	−0.49
Supramarginal_G	−0.46	0.021	−0.28	Supramarginal_G	−0.40	0.048	−0.38
Superior_Temporal_G	−0.42	0.039	−0.03	Superior_Temporal_G	−0.43	0.031	−0.17
Middle_Temporal_G	−0.43	0.035	−0.27	Middle_Temporal_G	−0.45	0.025	−0.15
Inferior_Temporal_G	−0.55	0.005	−0.42	Inferior_Temporal_G	−0.43	0.03	−0.1
Heschl_G	−0.53	0.006	−0.01				
Cerebellum_3	−0.45	0.042	−0.04				
Cerebellum_9	−0.41	0.045	−0.04				
Vermis_3 (non-hemispheric)	−0.41	0.046	−0.3				

The left-hemispheric structures of significance are more relevant in the context of right cerebral tumor groups. The correlations across left and right hemispheres are shown as *r*_*dis*_ for the discovery cohort, and *r*_*val*_ for the validation cohort, and the significance(*p* < 0.05) as *p*.

**Table 4:** AAL regions of the right cerebral tumor groups, within which MEDH was negatively correlated with long and short-term survival with subject age, and total tumor volume as confounders.

Left Hemisphere			Right Hemisphere		
Region	*r*_*dis*_	*p*	Region	*r*_*dis*_	*p*
Posterior_Cingulate_G	−0.380	0.080*	Parahippocampal_G	−0.45	0.033**
Hippocampus	−0.40	0.064*	Paracentral_Lobule	−0.36	−0.096*
Middle_Temporal_G	−0.39	0.065*	Middle_Temporal_G	−0.46	0.030**
Inferior_Temporal_G	−0.58	0.004**	Inferior_Temporal_G	−0.58	0.004**
Heschl_G	−0.37	0.088*			
Cerebellum_3	−0.44	0.038**			
Vermis_3 (non-hemispheric)	−0.42	0.047**			

The left-hemispheric structures of significance are more relevant for right cerebral tumor groups. The correlations for left and right hemispheres are shown as *r*_*dis*_ for the discovery set, with statistical signifcance *p* where ‘*’ is appended for *p* < 0.1 and ‘**’ for *p* < 0.05 respectively.

**Table 5:** AAL regions of the right cerebral tumor groups, within which the MEDH was negatively correlated with medium-term survival.

Left Hemisphere				Right Hemisphere			
Region	*r*_*dis*_	*p*	*r*_*val*_	Region	*r*_*dis*_	*p*	*r*_*val*_
Precentral_G	−0.49	0.046	−0.19	Precentral_G	−0.63	0.006	−0.28
Sup_Frontal_DorsoLateral	−0.55	0.023	−0.25	Sup_Frontal_DorsoLateral	−0.52	0.032	−0.06
Middle_Frontal_G	−0.54	0.026	−0.08	Middle_Frontal_G	−0.67	0.003	−0.17
Inf_Frontal_G_Tri_Part	−0.57	0.016	−0.21	Inf_Frontal_G_Tri_Part	−0.62	0.007	−0.03
Olfactory_Cortex	−0.53	0.028	−0.12	Olfactory_Cortex	−0.68	0.002	−0.21
Sup_Occipital_G	−0.60	0.010	−0.16	Sup_Occipital_G	−0.51	0.034	−0.46
Postcentral_G	−0.73	0.0008	−0.31	Postcentral_G	−0.63	0.006	−0.37
Inf_Parietal_G	−0.58	0.013	−0.03	Inf_Parietal_G	−0.58	0.018	−0.10
Sup_Frontal_G_Orbital_Part	−0.50	0.039	−0.09	Inf_Frontal_G_Opercular_Part	−0.66	0.003	−0.09
Insula	−0.52	0.031	−0.47	Sup_Frontal_G_Medial	−0.66	0.003	−0.18
Sup_Parietal_G	−0.63	0.003	−0.29	Gyrus_Rectus	−0.55	0.003	−0.14
Inf_Temporal_G	−0.60	0.01	−0.0005	Cuneus	−0.56	0.019	−0.14
Temporal_Pole_Middle_Temporal_G	−0.53	0.028	−0.13	Supramarginal_G	−0.54	0.025	−0.11
Vermis_3 (non-hemispheric)	−0.65	0.004	−0.83	Sup_Temporal_G	−0.52	0.032	−0.005
				Middle_Temporal_G	−0.48	0.048	−0.04

The correlations across left and right hemispheres are shown as *r*_*dis*_ for the discovery cohort, and *r*_*val*_ for the validation cohort, and the significance(*p* < 0.05) as *p*.

**Table 6:** AAL regions of the right cerebral tumor groups, within which the MEDH was negatively correlated with medium-term survival with subject age, and total tumor volume as confounders.

Left Hemisphere			Right Hemisphere		
Region	*r*_*dis*_	*p*	Region	*r*_*dis*_	*p*
Sup_Frontal_G_Orbital	−0.50	0.052*	Precentral_G	−0.61	0.014**
Sup_Frontal_G_DorsoLateral	−0.49	0.058*	Sup_Frontal_G_DorsoLateral	−0.47	0.070*
Middle_Frontal_G	−0.50	0.054*	Middle_Frontal_G	−0.66	0.007**
Middle_Frontal_G_Orbital_Part	−0.46	0.082*	Inf_Frontal_G_Opercular	−0.67	0.005**
Inf_Frontal_G_Tri_Part	−0.59	0.02**	Inf_Frontal_G_Tri_Part	−0.63	0.010**
Olfactory_Cortex	−0.56	0.028**	Olfactory_Cortex	−0.65	0.007**
Sup_Occipital_G	−0.56	0.026**	Sup_Occipital_G	−0.47	0.071*
Postcentral_G	−0.72	0.002**	Postcentral_G	−0.62	0.012**
Inf_Parietal_G	−0.55	0.032**	Inf_Parietal_G	−0.54	0.037**
Insula	−0.51	0.051*	Rolandic_Operculum	−0.48	0.068*
Sup_Parietal_G	−0.59	0.02**	Sup_Frontal_G_Medial	−0.66	0.006**
Temporal_Pole_Middle_Temporal_G	−0.46	0.08*	Gyrus_Rectus	−0.53	0.040**
Inf_Temporal_G	−0.57	0.024**	Cuneus	−0.54	0.036**
Vermis_3 (non-hemispheric)	−0.66	0.007**	Supramarginal_G	−0.62	0.012**
			Sup_Temporal_G	−0.50	0.055*
			Middle_Temporal_G	−0.44	0.097*

The correlations for left and right hemispheres are shown as *r*_*dis*_ for the discovery set, with statistical significance *p* where ‘*’ is appended for *p* < 0.1 and ‘**’ for *p* < 0.05 respectively.

### Functional areas associated with survival

**Figure 4:**
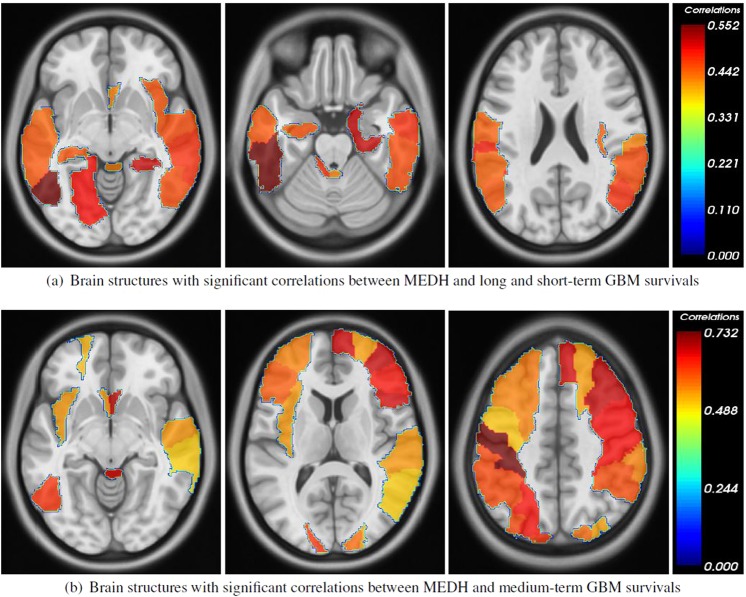
The AAL regions in which, the MEDH negatively correlated with survival with *p* < 0.05 for right-hemispheric tumor group (neurological view). The colormaps show the negative correlation values (shown as positive for easier representation). Structures on left-hemisphere may be more relevant as right-hemispheric structures may overlap with tumor within the group.

The MEDH, in AAL regions, due to the mass effect was associated with survival for right-hemispheric tumors. The specific functional areas within which MEDH was associated with survival in general were the precentral gyrus, olfactory cortex, superior and middle frontal gyri, parietal and the temporal gyri of the contralateral hemisphere. Increased MEDH within the left hippocampus and Heschl gyrus was also associated with poor survival. In all survival groups, a commonality was found in the association of MEDH in left postcentral gyrus and cerebellar vermis with survival. Tables 3 and 5 show detailed lists of all AAL regions in which, the MEDH negatively correlated with extreme ends (long and short-term) of survival and medium-term survival respectively with statistical significance (*p* < 0.05). The findings on the hold-out set (*r*_*val*_) demonstrated similar trends, except the following: Heschl gyrus in the left hemisphere and Insula in the right hemisphere exhibited lower correlation with survival days in the validation cohort, as compared to the discovery cohort. The inclusion of confounding variables i.e. age and total tumor volume at presentation did not however alter the correlated regions as shown in Tables 4 and 6 when compared to the results in Tables 3 and 5 respectively. The MEDH in inferior temporal gyri, parietal gyri, olfactory cortex, postcentral and occipital gyri of the contralateral left hemisphere maintained significance (*p* < 0.05), while those within the hippocampus and Heschl gyrus showed trend towards statistical significance (*p* < 0.1) with survival. Despite the fact that deformation in right-hemispheric structures may overlap with the tumor regions, for the sake of completeness we reported both right and left hemispheric structures in which significant correlations were observed. Nevertheless, we think the interpretation of MEDH in significantly correlated left-hemispheric structures is straightforward and relevant as the deformation in these regions is exclusive of tumor areas. Figure 4 shows the AAL regions color-coded with correlation values (*p* < 0.05) for both groups of survival. The concordance indices (CI) using the Cox proportional hazards model for AAL regions which were associated with survival (long + short-term for the right hemispheric lesion group), are reported in Table 7. While CI for most of the significant AAL regions was above 0.62, highest CI was observed for inferior temporal gyrus and Heschl gyrus with CI of 0.71, and 0.69 respectively. Further, when MEDH across all significant AAL regions were considered together in a multi-variate analysis, CI was found to be 0.76 for the left hemispheric tumors, and 0.72 for right hemispheric tumors respectively. Clinical parameters (KPS, IDH1, and MGMT status) were separately evaluated in a univariate fashion where each of the clinical variables was correlated against the survival days. Correlation coefficients and associated significance of each of the clinical parameters are given in Table 8. Interestingly, a combination of MEDH features from the significant AAL regions in the left-hemispheric tumors (Table 1) with the clinical variables (KPS, IDH1, MGMT) yielded a CI 0.82.

**Table 7:** The concordance indices (CI) using a proportional hazards model for AAL regions of right cerebral tumor groups within which MEDH was negatively correlated with long and short-term survival on the discovery cohort.

Left Hemisphere		Right Hemisphere	
Region	*CI*	Region	*CI*
Hippocampus	0.65	Olfactory Cortex	0.63
Lingual_G	0.67	Insula	0.65
Postcentral_G	0.65	Parahippocampal_G	0.67
Supramarginal_G	0.64	Supramarginal_G	0.63
Superior_Temporal_G	0.65	Superior_Temporal_G	0.65
Middle_Temporal_G	0.65	Middle_Temporal_G	0.66
**Inferior_Temporal_G**	0.71	Inferior_Temporal_G	0.65
**Heschl_G**	0.69		
Cerebellum_3	0.60		
Cerebellum_9	0.66		
Vermis_3 (non-hemispheric)	0.63		
Multi-variate (11 significant AAL regions)	**0.76**	Multi-variate (7 significant AAL regions)	0.72

Multi-variate analysis while accounting for MEDH values across all the significant AAL regions together yielded a higher concordance index, compared to a univariate analysis employing MEDH value for every single region separately.

**Table 8:** Statistical correlations and significance for IDH1 mutation status, MGMT methylation status, and KPS with the survival days, on the discovery cohort.

Variable	*r*_*dis*_	p
KPS	0.59	0.02
IDH1	0.48	0.11
MGMT	0.30	0.33

## Discussion

In this study we analyzed deformations in remote brain areas caused by tumor mass effect in GBM, and identified the functional areas in which the mass effect induced deformation heterogeneity (MEDH) was associated with overall survival. As observed in our study, significant association between MEDH within functional areas and survival of right cerebral tumors is yet another representation of GBM heterogeneity with regard to its manifestation of clinical symptoms based on tumor cerebral hemisphere. Our results are in concordance with the study by Liu *et al*.^11^ which demonstrated in a large study cohort that tumors in the right occipitotemporal periventricular white matter and periatrial areas were related to poor survival and prognosis. Other studies showed that patients with right hemispheric tumors manifested anxiety with significantly high mean anxiety scores^48,49^ and also had poor scores in facial recognition tests^50^. The tumors on right hemisphere being characteristically associated with poor survival is likely due to the location of eloquent functional areas in the left hemisphere for right-handed individuals^51^. This leads to delayed clinical presentation of symptoms in right-hemispheric tumors. The impact of mass effect in contralateral functional areas and its association with survival may be further corroborated by the fact that right hemispheric tumor volumes are higher^11^, showing a tendency of excessive infiltration and hence are difficult to resect, resulting in poor prognosis^52^. Interestingly, the patient demographics presented in Table 2 indeed shows more subjects with right hemispheric tumors with tumor crossings through corpus callosum and subfalcine herniation, and such effects were observed on the average tumor maps of Fig. 3(c,d). Table 2 however shows equal numbers of midline shifts in both right and left hemispheric tumor groups, this means other deformities related to mass effect are certainly prognostic factors of survival apart from midline shifts.

We observed the association of increased MEDH within temporal gyri with decreased survival in all groups (see Tables 3–6). A rare case was reported in^53^, where a patient with right-hemispheric GBM showed a crossed Wernicke’s aphasia. Many studies however have shown that of the 90% right-handed human population, about 96% exhibit left-brain language dominance along with motor behaviour^54–57^. A study by^58^ involving a large sample of left- and right-handed individuals showed ‘typical’ 78% left-brain language dominance in left-handers, and 88% left-brain language dominance in right-handers; a dominant ‘atypical’ right-hemisphere was shown for only 7% of left-handers and the other cases were ‘ambilateral’. Although the handedness data was unavailable in our study, the previous studies implicitly corroborate our findings related to the contralateral damage of right-hemispheric tumors as the primary auditory cortex on superior temporal gyrus (STG) and Wernicke’s area (the part adjacent to left STG) are located on the dominant left hemisphere. These brain areas are associated with the sensation of sound and language comprehension respectively^59,60^, and have been linked to decreased survival in patients with high-grade glioma^61^. The middle and inferior temporal gyri have been known to be involved in a number of cognitive processes and are the critical nodes of the brain’s language network. The middle temporal gyrus has been known to be involved in facial recognition process and assessing word meaning while reading. Inferior temporal gyrus is considered as one of the higher level ventral streams of visual processing associated with the representation of complex objects and facial recognition^62,63^. showed that verbal, visuospatial memory and word fluency were the most frequently affected functions before glioma surgery. While^32^ showed acquired postoperative language deficits were associated with decreased survival, although in most cases preoperative language deficits were worsened post GBM surgery. In addition to the temporal gyri, we found significant association of MEDH within the frontal gyri with survival (Tables 5 and 6). Filley *et al*.^64^ presented a model where three frontal lobe syndromes were associated with brain tumors in specific prefrontal areas. The loss of executive function was attributed to dorsolateral prefrontal damage, orbitofrontal damage caused disinhibition and impulsiveness, and medial frontal damage caused apathy or abulia^65,66^. It was also shown that patients with frontal tumors showed more executive dysfunction, apathy and disinhibition compared to non-frontal tumors^67^, and women with left frontal glioblastoma consistently showed significantly poor survival^68^. In our study, the primary sites of tumor impact were on the right hemisphere. The association of secondary contralateral frontal damage and survival therefore is not straightforward. It is however possible that the complex interactions between the cortical and subcortical damages through white matter pathways add to the neurological deficits and hence survival^34^.

The MEDH within the somatosensory cortex (postcentral gyrus), somatosensory association cortex (supramarginal gyrus), Heschl gyrus (auditory processing), primary motor cortex (precentral gyrus), and the olfactory cortex showed association with GBM survival. Although a direct relation of sensory and motor deficits with GBM survival has not been studied before, Chaicana *et al*.^33^ showed that despite extended tumor resection, the median survival was limited to only 7.9 months in 129 older patients, and all of them had preoperative sensory, motor and/or language deficits.

The MEDH within the hippocampus and cerebellum was particularly seen to be associated with poor survival (Tables 3 and 4). This is perhaps due to transtentorial herniation that is a known tumor mass effect associated with poor prognosis in GBM^69,70^. Hippocampus being related to memory functioning, long-term memory deficits were observed in malignant glioma^71^ and such neurocognitive deficits have been associated with survival^72^. Our results on the validation cohort were in concordance with the results obtained on the discovery set, except the following: Heschl gyrus in the left hemisphere and Insula in the right hemisphere exhibited significantly lower correlation with survival days in the validation cohort, as compared to the discovery cohort.

A recent radiomic study by Bakas *et al*.^36^ employed a total of 135 TCIA studies with a similar inclusion criteria as us, and publicly released a total of 102 of these studies and their associated radiomic features. Out of these 102 studies, while 93 cases were included in our cohort, 9 studies could not be included due to their diagnostic quality (as determined by our expert radiologists). We however did include an additional 45 studies from the TCIA cohort that followed our inclusion criteria, accounting to a total of 138 studies used for our analysis.

Another key difference across the studies used in^36^ and our cohort was in terms of the registration workflow. While our study employed an MNI512 atlas for registering the native MRI scans to a common space, the study in^36^, employed the SRI atlas. Hence, in order to compare the inter-reader variability across the segmentations curated in our study with those in^36^, we randomly identified n = 20 studies from our cohort and re-registered the segmentations provided in^36^ to the MNI space. A dice score of 0.76 ± 0.14 was obtained between the two segmentation sets, demonstrating a relatively high degree of agreement between the annotated regions of interest (combining enhancing, non-enhancing, necrotic core, and edema region).

Despite the clinical inferences relating to the functional areas being impacted by mass effect, this study has its own limitations. In the absence of neurocognitive functional measures, a part of our hypothesis that deformation due to mass effect in functional areas result in poor neurocognitive outcomes, could not be validated. Correlating the MEDH of known functional areas with the functional outcome measures would have further strengthened our scientific premise that mass effect in functional areas impact GBM survival. In future, such analysis may be performed with curated data from a clinical trial. Besides, MEDH features will be made publicly available for the research community, and can be used in the context of other brain tumors to study the impact of deformation.

The results reported are preliminary as this feasibility study was limited by a relatively small training and validation cohort. We think the MEDH analysis confounded on specific tumor locations (such as frontal, parietal, temporal) in conjunction with cerebral hemisphere would be more appropriate to clearly understand the relationship of GBM survival with mass effect. Using tumor location as a confounding factor would have allowed the analysis of MEDH and survival even in ipsilateral regions. A larger powered sample size with uniform distribution of tumors in different brain regions would be required for such an analysis. Besides, we have not studied the effects of deformation using other deformable registration methods such as FNIRT^47,73^ and NiftyReg^74,75^. These analyses will be part of future work.

In conclusion, this study has provided a method for the analysis of deformation due to mass effect (MEDH) within remote contralateral tumor structures. The analysis demonstrated association of specific remote functional areas with survival for the right-hemispheric tumors. To the best of our knowledge, this is the first study that aimed to study the impact of mass effect, which perhaps causes persistent neurological deficits, and hence may impact survival. However, in order to better understand the effect of MEDH in functional areas and its association to GBM survival, we will need to validate our findings as a part of a larger statistically powered multi-site study.

## Supplementary information

Supplementary Document

Data cohort
