# Supplementary material for: Mass Effect Deformation Heterogeneity (MEDH) on Gadolinium-contrast T1-weighted MRI is associated with decreased survival in patients with right cerebral hemisphere Glioblastoma: A feasibility study
Source: Sci Rep. 2019 Feb 4;9:1145. doi: 10.1038/s41598-018-37615-2 (PMC6362117; doi:10.1038/s41598-018-37615-2)
Supplement: Supplementary file 1 — Supplementary Document [file 41598_2018_37615_MOESM1_ESM.docx]

**Mass Effect Deformation Heterogeneity (MEDH) on T1-weighted MRI is associated with decreased survival in patients with right cerebral hemisphere Glioblastoma: A feasibility study**

Prateek Prasanna^1,*^, Jhimli Mitra^1,2,*^, Niha Beig^1^, Ameya Nayate^3^, Jay Patel^1^, Soumya

Ghose^1^, Rajat Thawani^1^, Sasan Partovi^3^, Anant Madabhushi^1^, and Pallavi Tiwari^1,+^

1Case Western Reserve University, Department of Biomedical Engineering, Cleveland, USA

2General Electric Global Research, New York, USA

3University Hospitals of Cleveland, Department of Radiology, Cleveland, USA

+pallavi.tiwari@case.edu

*Equal contribution

**Supplementary Material**


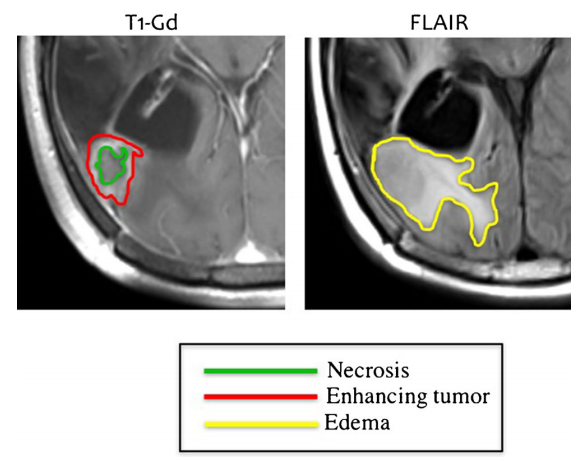


**Figure 1:** *Annotations of necrotic core and enhancing tumor are outlined in green and red, respectively as delineated by an expert on a representative gadolinium (Gd)-T1w MRI slice, while the annotations for the edema region as delineated on FLAIR are shown in yellow.*
